# A Review of Suicide Risk Assessment Tools and Their Measured Psychometric Properties in Korea

**DOI:** 10.3389/fpsyt.2021.679779

**Published:** 2021-06-22

**Authors:** In-Chul Baek, Soobin Jo, Eun Ji Kim, Ga Ryoung Lee, Dong Hun Lee, Hong Jin Jeon

**Affiliations:** ^1^Department of Psychiatry, Depression Center, Samsung Medical Center, Sungkyunkwan University School of Medicine, Seoul, South Korea; ^2^Korea Psychological Autopsy Center (KPAC), Seoul, South Korea; ^3^Department of Education, Traumatic Stress Center, Sungkyunkwan University College of Education, Seoul, South Korea; ^4^Department of Health Sciences & Technology, Department of Medical Device Management & Research, and Department of Clinical Research Design & Evaluation, Samsung Advanced Institute for Health Sciences & Technology (SAIHST), Sungkyunkwan University, Seoul, South Korea

**Keywords:** suicide, suicidal ideation, screening, assessment tools, validity, reliability

## Abstract

While there has been a slew of review studies on suicide measurement tools until now, there were not any reviews focusing on suicide assessment tools available in Korea. This review aimed to examine the psychometric properties of tools developed in Korea or the translated versions from the original tools in their foreign language and to identify potential improvements and supplements for these tools. A literature search was done using the Korean academic information search service, Research Information Service System, to identify the suicide measures to be included in this review. Abstracts were screened to identify which measures were used to assess suicide-related factors. Based on the established inclusion and exclusion criteria, 18 tools remained and we assessed their psychometric properties. The current review indicated several major findings. First, many of the tools did not report predictive validity and even those with predictive validity were based on past suicide attempts. Second, some of the tools overlooked the interactive component for the cause of suicide. In addition, information to supplement the self-reported and clinician-administered reports by collecting reports from the subjects' families and acquaintances is needed. It is also important to develop a screening tool that examines other aspects of an individual's personal life, including unemployment, bereavement, divorce, and childhood trauma. Moreover, tools that have been studied in more diverse groups of the population are needed to increase external validity. Finally, the linguistic translation of the tools into Korean needs to consider other cultural, social, and psychological factors of the sample of interest.

## Introduction

The overall suicide rate in South Korea began to rise in 1992. The trend was accelerated in 1998 when the International Monetary Fund (IMF) crisis occurred, and again in 2009, just after the global financial crisis. As of 2017, the suicide rate per 100,000 people was 24.3, which is the second-highest suicide rate among the OECD countries (1).

In addition, according to the results of an epidemiological survey of mental illness, 3.2% of people showed lifetime suicide attempts, and 1.1% had attempted suicide more than once (2). In this regard, it can be said that it is nationally important to appropriately assess suicide risk and the value of the tool treatment professionals have at hand.

The most common method of measuring suicide risk is to identify symptoms related to suicide risk through self-reported questionnaires. Suicide risk groups are usually screened this way, and experts interview the subjects in order to determine the severity of their suicidal ideation. In this case, the risk of suicide is predicted by excluding the intervention of each expert's ability or the expert's subjective judgment as much as possible, and by using a structured interview tool for efficiency.

There have been many review studies on these suicide assessment tools. Goldston (3) reviewed most of these tools in children and adolescents. Furthermore, Brown (4) reviewed extensively and in detail the suicide measurement tools for adults composed in English, analyzing both their reliability and validity. Perlman et al. (5) reviewed suicide measurement tools that can be used in clinical settings, and Batterham et al. (6) reviewed the suicide risk self-reporting tool for the general adult population according to a systematic review method. Park et al. (7) reviewed the measurement tools for children, adolescents, and adults focusing on suicide predictive power. However, there were no cases of reviews that focused on suicide assessment tools available specifically in Korea. Therefore, the purpose of this study was to examine the psychometric properties of tools developed in Korea, or the translated versions of the originals in their foreign languages, in order to identify potential improvements and supplements for said tools.

## Methods

### Selection of Measures

A literature search was done using a Korean database platform, Research Information Service System (RISS), to identify suicide measures and to include them in this review. Search terms used included: suicid^*^ AND valid^*^. The abstracts were screened to identify measures that were used to assess suicide-related factors including suicidal thoughts, behaviors, intention, attitudes, hopelessness, and their severity. Additional measures were sourced from the reference list.

Measures containing two or more items that assessed suicidal thoughts, behaviors, or suicide-related factors and yielded quantitative data were retained. These inclusion criteria were established as they aligned with the variables of interest of this review and were evidence-based reports. However, measures were excluded from this review if they primarily assessed non-suicidal self-harm, gatekeeper's attitude toward suicide, depression, suicide stigma, general mental health, were not validated with a paper published in at least one peer-reviewed journal, or any sign language versions. Papers with non-suicidal self-harm, gatekeeper's attitude toward suicide, depression, suicide stigma, general mental health were part of the exclusion criteria as these did not specifically assess the participants' suicidal construct which is the primary objective of this review paper. Reviews that were not validated in a peer-reviewed journal were excluded because these reviews may not have been reviewed by experts, which may result in low validity of the findings. Finally, the sign language versions were not included because this review aimed to focus on assessments that are administered to the general population.

### Evaluation of the Quality of Measures

Measures were evaluated based on the psychometric properties demonstrated in at least one study, based on the following criteria:

a) Internal consistency—determines whether a measure's items measure the same domain;
b) Test-retest reliability—assesses the consistency of results at two different time points;
c) Concurrent validity—demonstrates the theoretical structure of scores on the measure, their ability to discriminate, and how well they correlate with related variables;
d) Construct Validity—demonstrates the extent to which scores on a measure related to scores on a similar measure at the same point in time;
e) Predictive validity—how accurately the measure predicts the target variable.

## Results

### Identification of Measures

**Figure 1:**
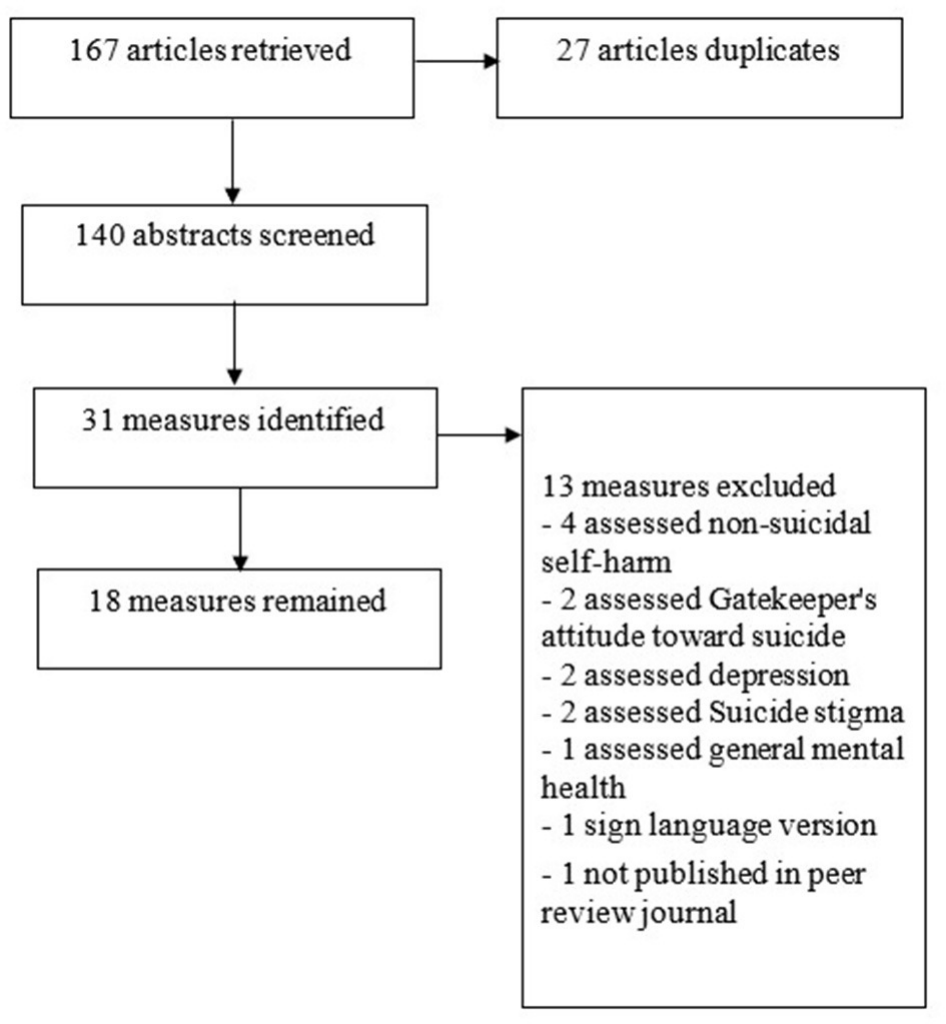
Flow diagram of the systematic review to identify suicide measures.

The flow of the literature search and review is shown in Figure 1. From 140 abstracts, 31 measures were identified. However, two of the papers only measured depression, two measured gatekeeper's (people who identify individuals with possible suicidal risks) attitude of gatekeepers toward suicide, two measured social stigmas toward suicide, four measured non-suicidal self-harm, one measured sign language-version, one measured general mental health, and one was not published in a peer-review journal. Finally, the 18 measures that were included in this review were: Columbia Suicide Severity Rating Scale (CSSRS), Beck Scale for Suicide Ideation (BSSI), Suicide Probability Scale (SPS), Screening for Depression and Thoughts of Suicide, Beck Hopelessness Scale (BHS), Nurses' Global Assessment of Suicide Risk (NGASR), Suicide Risk Screening Scale for Incarcerated Offenders (SRSSIO), Suicidal Imagery Questionnaire (SIQ), Depressive Symptom Inventory-Suicidality Subscale, Suicide Risk Scale for Medical Inpatients, Korean Geriatric Suicidal Risk Scale, Suicidal Dangerousness Scale for Military Soldiers, Reason For Living (RFL), Reasons for Living for Young Adults (RFL-YA), College Student Reasons for Living Inventory (CSRLI), Reasons for Living Inventory for Adolescents (RFL-A), Measurement of Suicidal Protection, and Suicide Resilience Inventory (SRI) (Table 1).

**Table 1:** Characteristics and psychometric properties of suicide measures.

**Measure**	**Mode of**		**Items**	**Sample**	**Reliability**		**Validity**		
	**administration**								
	**Self-report**	**Interview**			**Internal consistency**	**Test–retest** ** (inter-rater)**	**Concurrent**	**Construct**	**Predictive**
CSSRS (8)		√	10	100 MDD patients (31 suicide attempters, 69 non-suicide risk group)	α range 0.62–0.88	Not reported	Correlated with SSI (*r* range 0.55–0.70), Hamilton-depression scale-suicide item *(r* range 0.62–0.63), BDI-suicide item (*r* range 0.44–0.61)	Two factors: passive suicide idea without intention, active suicide idea with intention	58.6% Sensitivity and 79.6% specificity for suicide attempt
CSSRS (9)		√	10	31 inpatient, diagnosed with alcohol dependent disorder	α = 0.90	Not reported	Correlated with SSI (*r* = 0.43–0.65), BDI-Suicide Scale (*r* = 0.50–0.66), Beck hopelessness Scale (*r* = 0.46–0.47)	Not reported	Not reported
Beck Scale for Suicide Ideation (10)			21	1,241 undergraduate students	α = 0.74		Correlated with SBQ-R (*r =* 0.62), CES-D (*r =* 0.40)	Two factors: active suicide idea, ambivalent attitude for suicide	Not reported
Beck Scale for Suicide Ideation (11)	√		21	2,392 community sample	*0.90*	Not reported	Correlated with BDI-II (*r =* 0.68), Beck Anxiety Inventory (*r =* 0.52), Beck Hopelessness Scale (*r =* 0.47)	Two factors: motivation, preparation	Not reported
SPS (12)	√		31	792 middle school, high school student	0.70 Subscale range 0.65–0.80	Half reliability 0.69–0.71	Correlated with Rosenberg self-esteem scale (*r =* 0.72), Subscale Hopelessness correlated with Hopelessness scale for children (*r =* 0.27)	Four factors: hopelessness, suicidal ideation, negative self-evaluation, hostility	Not reported
Screening for Depression and Thoughts of suicide (13)		√	2	325 outpatients	Not reported	Not reported	Not reported	Not reported	60.9% sensitivity and 83.3% specificity for lifetime suicide attempt
BHS (14)	√		20	1,022 community sample	A = 0.85	0.86 over a week	Correlated with PHQ-9 (*r =* 0.46), STAI-S (*r =* 0.51), STAI-T (*r =* 0.54)	Three factors: hopefulness, giving up, future expectations	Not reported
NGASR (15)		√	15	92 psychiatric inpatients	Not reported	Inter-rater Kappa = 0.89	Correlated with Evaluation of Suicide Risk (Jonckheere-Terpstra Test J = 4.69)	Six factors: hopelessness, suicidal desire, psychosis, relationship breakdown, withdrawal, family history	Not reported
SRSSIO (16)	√		32	459 incarcerated offenders	α = 0.97 Subfactor 0.78–0.95	Not reported	Correlated with Reynolds Suicidal ideation scale (*r =* 0.72), BDI (*r =* 0.72), Barratt impulsivity scale II (*r =* 0.38), Hopelessness depression scale (*r =* 0.76), Optimism (*r =* −0.50)	Five factors: hopelessness about the future, cognitive and behavioral impulses, suicide incidents, depression and helplessness in daily life, self-harm thoughts	Cut-off 16 (sensitivity 0.91, specificity 0.81)
Suicidal Imagery Questionnaire (17)	√		10	365 adults 433 adults (online survey)	0.94 Subfactor 0.93–0.94	Over 2 weeks (*r =* 0.88)	Correlated with CSSRS – screening version (*r =* 0.61), Spontaneous Use of Imagery Scale (*r =* 0.22)	Two factors: spontaneous suicidal imagery, intrusive suicidal imagery	Not reported
Self-report Depressive Symptom Inventory-Suicidality Subscale (18)	√		4	554 undergraduate students	0.93	Not reported	Correlated with Beck SSI (*r =* 0.70), BDI (*r =* 0.57), Insomnia Severity Index (*r =* 0.27)	One factor	Not reported
Suicide Risk Scale for Medical Inpatients (19)	√		7	100 psychiatric inpatients	0.91	*R =* 0.64	Correlated with HADS (*r =* 0.73), Beck SSI (*r =* 0.58), BHS (*r =* 0.36)	One factor	Cut-off 5, 71.4% sensitivity, 75.6% specificity
Korean Geriatric Suicidal Risk Scale (20)	√	√	24	312 (52 suicide attempter)	Kuder-Richardson-21 = 0.79	33 over 2 weeks (*r =* 0.92)	Correlated with NGASR (*r =* 0.76), SIS [suicide ideation scale; (21)] (*r =* 0.62)	Not reported	Past suicide attempter Cut-off 11, 93.9% sensitivity, 75.7% specificity, 43.1%
Suicidal Dangerousness Scale for Military Soldiers (22)	√		20	1,091 soldiers	α = 0.85 Subfactors (*r =* 69 −0.92)	Not reported	Correlated with depression (*r =* 0.63) Hopelessness (*r =* 0.60), self-esteem (*r =* −0.57)	Four factors: Experience of suicide attempt, suicidal desire relief, suicidal plan concealment, motive for suicidal ideation	Not reported
RFL (23)	√		48	320 adults Community population (G. W. 34)	Subscales α range 0.68–0.95	Not reported	Survival and Coping Subscales Correlated with SSI (*r =* −0.24), Optimism (*r =* 0.60)	Four factors: Survival and coping beliefs, fear of suicide and social disapproval, responsibilities to family and child-related concerns, future expectations	Not reported
KRFL-YA Reasons for Living for Young Adults (24)	√		32	545 young adults (suicide attempter 71)	0.95 Subscale 0.82–0.92	Not reported	Correlated with SSI (*r =* −0.42), Optimism (Reevaluation of Life Orientation Test) (*r =* 0.37)	Five factors: Family relations, positive self-evaluation, coping beliefs, relationship with peers, future expectations	Not reported
The College Student Reasons for Living Inventory (25)			46	289 undergraduates					
College Student Reasons for Living Inventory (26)	√		49	445 undergraduates	Subscale 0.76–0.96	Not reported	Correlated with Family Hardiness Indexes (*r =* 0.59), SSI (*r =* −0.72)	Five factors: Survival/coping beliefs and future expectations, responsibilities to family and peers, fear of social disapproval, fear of suicide, moral objections Discriminated	Not reported
Korean version of the Reasons for Living Inventory for Adolescents, KRFL- A (27)	√		32	751 high school students (male 291, female 460)	0.98	Over 1 month (*r =* 0.85)	SSI (*r =* −0.34), Beck Hopelessness scale (*r =* −0.46)	Three factors: Peer acceptance, self-acceptance and optimism about the future, family alliance, fear of suicide	Not reported
Measurement of Suicidal Protection (MSP) (28)	√		26	330 high school students	0.93 Subfactors 0.72–0.86	Not reported	Correlated with the reasons for living inventory for adolescents (RFL-A) (*r =* 0.83)	Six factors: Fear of suicide, self-esteem, emotion regulation, support from others, support from family, and school life	Not reported
Suicide Resilience Inventory-Korean Version (SRI-K) (29)	√		25	278 undergraduate students	0.94 Subfactors 0.88–0.93	Not reported	Not reported	Three factors: Internal protective, external protective, emotional stability	Not reported

*MDD, Major depressive disorder; SSI, Scale for Suicidal Ideation; BDI, Beck Depression Inventory; CSSRS, Columbia Suicide Severity Rating Scale*.

### Description of Measurement Tools and Their Psychometric Characteristics

#### Columbia Suicide Severity Rating Scale (CSSRS)

This is a scale developed by Posner et al. (30). This evaluation tool considers the clinical symptoms and the risk factors related to suicide through semi-structured interviews. The subscales of CSSRS consist of the severity of the suicidal ideation, the intensity of the suicidal ideation, suicidal behavior, and fatality of suicidal behavior. The items for the severity and the intensity of suicidal incidents are on a 5-point ranking scale while those for the suicidal behavior are on a naming scale and those for the fatality of the suicidal behavior are on a 6-point scale. The Korean version was standardized by Jang et al. (8) with a highly internally consistent alpha value, but the test-retest reliability was not reported. As this validation was an exploratory factor analysis, further validation is needed whether the identified factors can be applied to other clinical groups. Furthermore, among patients with major depressive disorder, comorbidity with anxiety disorder and personality disorders increases the risk of suicide accidents or suicide attempts. This validation did not rule out such coexisting disorders and therefore requires careful interpretation of the results. Pai et al. (9) also reported a high internal consistency alpha value in CSSRS, but the construct and predictive validities were not reported, nor was the test-retest reliability.

#### Beck Scale for Suicide Ideation (BSSI)

This is a self-report questionnaire developed by Beck et al. (31) that consists of a total of 21 questions attempting to measure suicidality and the severity thereof. The content of the questions covers several topics such as the desire for life and death, the frequency of suicide incidents, the perceived sense of control to commit suicide, and the degree of actual preparation. Based on the participants' experience of the past weeks, a 3-point Likert scale (0–2 points) was used. Questions 1–5 are screening questions, asking whether they have an active or passive desire for suicide, in which three items evaluate the participants' desire to live or die and two items evaluate their desire to attempt suicide. If they show any suicidal desire, then the remaining items of the questionnaire are administered. In addition, items 20 and 21 ask about the number of suicide attempts in the past and the severity of suicidal intentions at the time of the last suicide attempt, neither of which are included in the total score. As a result, the total score of the questionnaire ranges from 0 to 38 points. In the research by Lee and Kwon (10) and Choi et al. (11), the internal consistency of the BSSI is high while the test-retest reliability and predictive validity were not calculated. The validation by Choi et al. (11) suggests to conduct additional validation with a clinical group and with more diverse samples that include teenagers and the elderly.

#### SPS (Suicide Probability Scale)-Adolescent

The SPS is a self-report scale developed by Cull and Gill (32) that predicts suicidal behavior in adults and adolescents over the age of 14 and consists of 36 questions. It consists of four clinical subscales: 12 questions for hopelessness, 8 questions for suicidal ideation, 9 questions for negative self-evaluation, and 7 questions for hostility. Based on the fact that the tool is inappropriate for adolescents (33, 34), Go et al. (12) developed and standardized the tool for the adolescent population. The value of half reliability was reported instead of the test-retest reliability. The predictive validity was once again not reported. Furthermore, the authors suggest that additional studies should be conducted to identify cut-off points to identify depression risk groups among adolescents.

#### Screening for Depression and Thoughts of Suicide

This is a tool developed by mental health social workers in the Norton Sound area of Alaska, USA, where there is a high suicide rate. This tool features concise screening questions along with a well-written policy. It consists of two questions: “In the past few weeks, have you ever been sad or desperate?” and “Did you have any plans or plans to harm yourself?.” Kim et al. (13) converted the tool to a Korean version, but the reliability, convergence validity, and construct validity were not reported. The predictive validity was reported but limited as it was based on analyzing whether past suicide attempts were properly classified. The research also indicated that there is a limitation of generalizability as the study was conducted at only one university hospital, one psychiatric hospital, and one health center.

#### Beck Hopelessness Scale (BHS)

The Beck Hopelessness Scale, developed by Beck et al. (35), is a 20-item self-report scale that measures perceived negative attitudes toward the future (pessimism) in adults. Recalling the past week for each question, subjects were asked to answer ‘yes' or ‘no' to the questions regarding their attitudes. Of the 20 questions, 9 are composed of reverse questions, and if there are multiple responses, the score is coded in the direction that indicates higher severity. The total score is calculated by summing the scores of each question, and the higher the score the greater the sense of hopelessness. In the Korean version, Kim et al. (14) validated the adaptation of this tool. The internal and test-retest reliability were reported as good, and the appropriate configuration validity was shown in a three-factor model. A significant correlation was reported with depression and anxiety scales, but the correlation with other suicide scales was not reported. The predictive validity was not reported either. Although the study validated this tool among general adult groups, further validation with clinical groups is needed to analyze the clinical efficacy and effectiveness of the assessment tool.

#### Nurses' Global Assessment of Suicide Risk (NGASR)

Cutcliffe and Barker (36) developed the Nurses' Global Assessment of Suicide Risk (NGASR) to help assess the suicide risk of novice medical personnel in the psychiatric ward. The NGASR assesses suicidal behavior along with suicide incidents, which is evaluated by medical staff members but can also be evaluated by nurses with limited experience in suicide assessment. It has been translated and applied in Germany, China, and the Netherlands, and has been standardized in Korea by Shin et al. (15). The test-retest reliability, concurrent validity, and construct validity were reported, but the internal consistency and predictive validity were omitted. In addition, as the validation of this tool in Korea was done only in one hospital setting, it is important to validate this tool in other clinical settings to further generalize the results to other populations.

#### Suicide Risk Screening Scale for Incarcerated Offenders (SRSSIO)

Song and Lee (16) selected variables that are highly related to suicide, taking into account the characteristics of the inmates, and developed this tool to screen the inmates' suicide risk. The results from the preliminary questionnaire consisted of a dichotomous response scale—yes/no, in which participants showed the tendency to choose one of the options inattentively. Therefore, the scale of the SRSSIO is reorganized to a 4-point Likert scale. Inmates do not tend to respond honestly due to social desirability, which is a phenomenon when participants respond to seem as socially desirable rather than responding to reflect their true thoughts or feelings (37). Therefore, the items on the demographic information to identify their identities were excluded. The test-retest reliability was not reported in the standardized study. When a total score of 16 points was set as the cut-off score, a sensitivity of 0.91 and a specificity of 0.81 were reported, but there was a limitation in calculating the predictive validity based on past suicide attempts. In addition, the results of the validation may have been affected due to the social desirability bias of the participants. Finally, due to methodological issues, the sample size was not diverse in terms of variables related to mental health and coping skills among participants, which future research can consider.

#### Suicidal Imagery Questionnaire

Ko and You (17) developed a suicide image scale consisting of 13 questions. It consisted of 6 questions about spontaneous suicidal imagery, which is the experience of intentionally thinking about death, and 4 questions about invasive suicidal imagery, which asks about sudden and repetitive imagery experiences related to suicide. Each question was structured to select from a scale ranging from 0 (not at all, never experienced) to 4 (very often) for the degree that is most similar to the subjects' own experiences over the past 6 months. Most of the reliability and validity tests were adequate, but the predictive validity was not reported. A limitation of this validation is that further validation is needed to increase the generalizability to clinical populations, older age groups, and both genders as the sample consisted of women and young adults of non-clinical groups. Furthermore, the suicidal imagery scale developed in this study did not include questions about the vividness and immersion of such images, which are factors that can increase the severity of suicidal images.

#### Depressive Symptom Inventory-Suicidality Subscale

The DSI-SS is a subscale of the Hopelessness Depression Symptom Questionnaire. It was developed as a brief screening tool for suicide risk in general health settings. Joiner et al. (38) standardized the tool, and Suh et al. (18) standardized the Korean version of it. The DSI-SS consists of 4 items assessing the frequency and intensity of suicidal ideation, formulation of plans for suicide, controllability of suicidal thoughts, and suicide-related impulses during the previous 2 weeks. Each item is rated on a scale from 0 to 3 (total score range 0–12), with higher scores reflecting a higher severity of suicidal ideation. Suh et al. (18) reported a one-factor structure consistent with the original scale. The test-retest reliability and predictive validity were not reported due to the cross-sectional study design. Some limitations of the validation by Suh et al. (18) include limited generalizability due to a small sample in the study and also the use of limited constructs to identify convergent validity.

#### Suicide Risk Scale for Medical Inpatients

This tool was developed by Park et al. (19) to screen for suicide risk in clinical patients. It consists of 7 questions on a scale of 0 to 3 where 0 indicates strongly disagree and 3 indicates strongly agree. Both the reliability and validity have been reported, however, the sample size in the study was 100, which is insufficient for multivariate analysis. In addition, the correlation coefficient with HADS, which measures depression, was larger than that with BSSI, which measures suicidal thoughts. Therefore, the concept measured by the tool seems to be depression. A few limitations were presented in the study. First, the validity of the tool was examined by investigating the correlation with previously validated scales rather than the correlation with actual suicide attempts, which does not guarantee that this assessment can predict actual suicide attempts. Finally, it is also important to note that the calculation of cut-off scores was based on the BHS score rather than other suicide risk assessment tools.

#### Korean Geriatric Suicidal Risk Scale

This tool was developed by Lee and Kim (20) to detect high-risk suicide groups earlier in an effort to prevent suicide among the elderly over 65 years living in and can be conveniently administered by the medical personnel or by the general public. It consists of 24 questions, with 1 being yes and 0 being no, and the total score ranged from 0 to 24. The reliability and concurrent validity were appropriate, but constituent validity such as factor validity was not presented. When the cut-off score was set as the total score of 11 points, the positive predictive power was 43.1% and the negative predictive power was 98.5%. Unfortunately, these results are once again limited as they are based on past suicide attempts.

#### Suicidal Dangerousness Scale for Military Soldiers

This tool was developed by Sim et al. (22) to measure the suicide risk among soldiers. It consists of 20 questions with 4 factors, including experience in attempting suicide, suicide desire rescue clause, suicide plan concealment, and suicidal thought motivation. A 5-point scale (1: not at all and 5: very much) is used to assess how well each question describes his or her usual response style, and the higher the score, the higher the degree of suicide risk. The predictive validity was not reported, and a retest was performed, but the correlation coefficient of the test-retest reliability result was not reported because only the Cronbach's alpha value was calculated. In addition, the authors noted that suicide is highly influenced by various factors in the environment. Hence, quantitative research alone is limited to investigate the suicidal risks in depth. Therefore, a more accurate assessment of suicide risk may be possible by using both quantitative and qualitative measures. Furthermore, the study compared the correlation between depression, despair, self-esteem scales to investigate the criterion validity. However, the procedure to find the criterion validity was not verified. Hence, additional studies should compare the scales developed in this study with other existing suicidal ideation measures validated in the country.

#### Reason for Living

This is a tool developed by Linehan et al. (39) to investigate the belief system of a person at risk of suicide but without suicide attempts. It is composed of 48 items with a 6-point Likert scale and divided into 6 subcategories: survival and coping beliefs, family responsibility, concern for children, fear of suicide, fear of social criticism, and moral taboos. The total score ranged from 48 points to 288 points where a higher score was interpreted as having more reasons to not commit suicide. Lee et al. (23) validated the adaptation of the Korean version, though it was reported with four factors unlike the six factors of the original scale. On the original scale, the test-retest reliability at 3 week intervals was reported as satisfactory at a level of 0.75–0.85, but neither the test-retest reliability nor the predictive validity of the Korean version were not reported.

#### Reasons for Living for Young Adults

Gutierrez et al. (40) developed the Reasons for Living for Young Adults (RFL-YA) scale, a new scale of RFL that is specialized for college-aged youth. Cha and Kim (24) validated the Korean version adaptation. Like the original scale, it consisted of 32 questions, and a 6-point Likert scale was used, ranging from 1 point: not at all important to 6 points: very important. Cha and Kim (24) reported that the internal consistency, concurrent validity, and construct validity were appropriate, but the test-retest reliability and predictive validity were not reported. It is also important to note that validation of this tool sampled young adults specifically in a region of Korea and the metropolitan area. Therefore, future studies should target groups from other regions to establish a representative population of the country. Furthermore, the difference between social and cultural background can yield different opinions on the reason for living due to these factors affecting the value of life. Hence, it is required to conduct more research on cultural and social factors in the scale.

#### College Student Reasons for Living Inventory

Westefeld et al. (25) developed CSRLI to fully reflect the specific psychosocial factors of college students. Park and Ahn (26) analyzed 46 CSRLI questions and 113 reasons collected by them and then developed a Korean version of the tool consisting of 49 questions. It consisted of 5 factors with a Likert scale that ranged from 1 to 6 points. This validation proposed a few limitations. First, due to the nature of exploratory research, additional psychometric verification is needed for reliability. Furthermore, as this validation was focused on examining protective factors against suicide and used to screen non-clinical university students, additional studies are required to validate whether this tool can bring about the same results in a clinical group of university students. Meanwhile, Park and Ahn (26) reported a good level of internal consistency and concurrent validity but did not report the test-retest reliability and predictive validity.

#### Reasons for Living Inventory for Adolescents

Linehan et al. (39) and Osman et al. (41) developed a youth scale based on an adult life reason scale. It is aimed at adolescents aged from 14 to 18 and the subjects answer how important each question is as a reason for not committing suicide on a 6-point Likert scale (1 point being not at all important, 6 points being very important). Lee et al. (27) validated the translation into the Korean adaptation but the predictive validity was not reported. A few limitations of this validation include only studying students in one grade in high school, not including comparisons to other positivity-related measures, and failing to present a cut-off value to measure the risk of suicide among the participants.

#### Measurement of Suicidal Protection

Park and Yang (28) applied an ecological model to identify and measure the properties of suicide protection factors among Korean high school students. The answers fall on a 6-point Likert scale (1 point: not at all, 6 points: very yes), and 2 of the total 26 questions were coded as reverse questions. The intrinsic consistency, convergence validity, and construct validity were reported to be appropriate, but the test-retest reliability and predictive validity were not reported. This validation contains a few limitations. First, although the study analyzed variables at different levels such as individual, inter-individual, and organizational factors, it did not analyze community factors that refer to social norms and policies. Furthermore, this study contains groups of high school students; however, applying this tool to students who attend specialized schools or who are exposed to a high level of stress needs to be evaluated.

#### Suicide Resilience Inventory-Korean Version (SRI-K)

The SRI was developed by Osman et al. (42) to evaluate protective factors that can overcome suicide crises among adolescents and college students. It consists of a total of 25 questions and is composed of subscales of internal protection, external protection, and emotional stability. The internal protective scale is composed of 9 items that measure beliefs or feelings about one's self and life satisfaction. The external protective scale is composed of 8 questions that measure the ability of an individual to find external resources that he or she perceives as available when faced with difficulties or suicide. In addition, emotional stability, which is based on a constructed scale, measures positive beliefs that can control suicide-related thoughts and behaviors when faced with emotional or psychological stress events such as depression or rejection in interpersonal relationships. Each question is on a 6-point scale (1 point: not very, 6 points: very much), and the higher the score is, the lower the suicide risk is. This tool was translated and standardized by Noh et al. (29), and it was confirmed that the same three correlated factors, internal protective, emotional stability, and external protective were suitable for the Korean version as it was for the original scale. Reports on the test-retest reliability, convergence validity, and predictive validity remain to be seen, and thus far only the internal consistency and construct validity have been reported. Moreover, this study has the limitation of using convenience sampling which does not contain the target population that includes different genders, ages, and regions. Finally, this validation excluded six questions out of the original 25 questions due to low factor loading from the factor analysis, and further validation of these excluded variables is needed.

## Discussion

As we have seen so far, many tools have been used to screen suicide risk groups or to predict suicide. Among the variety of depression screening tools, the Columbia Suicide Severity Rating Scale (C-SSRS) and Beck's Scale for Suicide Ideation (BSSI) are the most commonly used. The original version of both tools can be administered to a wide range of populations including adolescents, adults, inpatients, and outpatients, and enables for cross-cultural adaptation (43). There are several underlying reasons for their common usage. First, the original version of C-SSRS displays a very high level (100%) of both sensitivity and specificity for correctly classifying both interrupted and actual lifetime suicide attempts (30). In terms of the Korean version, this tool is one of the few tools with a reported predictive validity, with 58.6% sensitivity and 79.6% specificity for suicide attempts. In addition, the Korean version of both the C-SSRS and BSSI shows a medium to an increased level of internal consistency (Cronbach's coefficient of 0.62 to 0.90) compared to those screening questionnaires that are available for different populations. C-SSRS and BSSI have expanded their validation in the adult population to other population groups, such as inpatients diagnosed with alcohol dependence disorder and undergraduate students. Whether C-SSRS can be administered to a younger group like BSSI remains to be investigated.

A key difference between C-SSRS and BSSI lies in the construct validity. C-SSRS measures passive suicidal ideas without intention and active suicide ideas with intention, whereas BSSI for undergraduate students measures not only active suicidal ideas but also ambivalent attitudes toward suicide. Meanwhile, the BSSI version for the adult population measures motivation and preparation. It has also been reported that BSSI is more commonly used for assessing patients already at risk of suicide, whereas C-SSRS can also be used for assessing patients who are not at risk of suicide and rather examines the potential future risk of suicide (44). Although both tools are available in many different languages and have been cross-culturally validated, it was found that C-SSRS was more accessible than BSSI due to its online availability. It is also worth noting that BSSI is a self-reported version and therefore is easier to administer than the C-SSRS in which the questions are formulated for an interview structure (44).

Although most of the tools show statistically appropriate reliability and concurrent validity, there are some more considerations in selecting suicide risk. First, there were many cases where the predictive validity was not reported in the process of tool standardization. Even in the cases of CSSRS, Screening for Depression and Thoughts of Suicide, SRSSIO, Suicide Risk Scale for Medical Inpatients, and the Korean Geriatric Suicidal Risk Scale, which have reported predicted validities, said validity was not analyzed based on actual suicide attempts or deaths, but rather on past suicide attempts. Although past suicidal thoughts and attempts can be used as a reference for predicting future suicidal risks, it is important to consider any changes that may influence one's internal state which then changes future suicidal thoughts attempts. Therefore, it is unreasonable to say that these tools were practically examined to determine whether they predict future suicide attempts or suicide completion. To further validate these models, it is necessary to construct a standardized longitudinal study and collect data related to suicide completion or attempts after measurement to better derive predictive validity. In considering predictive validity, it is necessary to consider the fact that is it difficult to have high predictive validity in and of itself because the base rate of suicide completion is low. Lange and Lippa (45) and Belsher et al. (46) showed that when the base rate is low, positive predictive power (PPP) is inevitably low, whereas when the base rate is high, the negative predictive power (NPP) is inevitably high. In the case of suicide completion, the base rate is low, but a dilemma arises in that PPP is more important than NPP for suicide prevention. No matter how well a tool is constructed, it is difficult to increase PPP. Nevertheless, an analysis of how long the tool predicts suicide attempts must be done to determine how useful it is for suicide screening.

Second, most of the suicide measures discussed above have limitations in that they measure only some of the factors related to suicide, such as suicidal behavior and suicidal thoughts or protective factors. It is known that suicide is caused by a complex interaction of various proximal and distal factors (47, 48). The vulnerability stress model that emerged in the 1950s explained that the preceding vulnerability factors were activated by stress and caused negative results such as psychiatric disorders which are often determined by the individual genetic makeup. In the integrated motivational-volitional model, which has further elaborated on this phenomenon, it is reported that problem-solving skills, social support factors, and cognitive biases act as mediating factors until stress experiences influence these aspects and eventually lead to suicidal behavior. When developing such suicidal assessment scales, it is necessary to consider the proximal and distal factors mentioned in the foregoing model that influence suicide incidents and suicidal behavior. According to the research results of O'Connor and Nock (47) and Turecki (48), when designing a tool to predict suicidal behavior, it is necessary to consider the following factors: personality and individual differences, cognitive factors, social factors, negative life events, psychiatric disorder, and physical disorders.

In addition to the aforementioned factors, other aspects of one's life can be considered to elucidate the complex interaction of proximal and distal factors that influence suicide such as unemployment, divorce, bereavement, and childhood trauma. Currently, there is an insufficient number of studies on screening tools that assess these factors of trauma among the Korean population. It has been reported that such factors are considered a large part of the etiology of depression and suicide (49). A plethora of studies have investigated the relationship between these factors and depression risk, however, there is a lack of validated screening tools for such factors in the translated version of the original version. For instance, although there have been previous studies on the relationship between unemployment and depression (50–52), the process of screening participants' employment status stems from either self-structured questionnaires or datasets from third parties such as the Korea Employment Information Service. There have also been studies to assess the validity of childhood adversity questionnaires, such as the Traumatic Event Screening Inventory and the Childhood Trauma Questionnaire, to identify childhood trauma (53, 54). Although the Childhood Trauma Questionnaire has been validated in Korean, these tools are administered via interview or self-report. As mentioned previously, there needs to be supplemental information collected from subjects' families and acquaintances to correct for recall bias as shown in a previous study (55). It is, therefore, imperative to either use one of the few validated tools in Korean as a supplement to assessments that measures suicidal risk or develop a tool to screen suicide risk factors in relation to depression, especially among the Korean population. Such developments to validate screening tools on personal aspects could enable future research to identify the relationships between changes in personal aspects and depression risk more accurately.

Third, there is a need for a tool that complements the strengths and the weaknesses of self-reports and clinician-administered reports, and that obtains confirmatory information from subjects' families and acquaintances. Self-reporting tools have the advantage of being easy to implement and report more recent suicide incidents but have generally failed to predict suicide-related outcomes in previous studies. Problems such as a poor understanding of the questions may arise, and the level of education may further influence the interpretation of these questions (56). In addition, the subject can also simply deny their suicide risks despite having a suicide incident and plan. In the case of the interview evaluation formula, although it is generally less accurate than the self-reported evaluation of recent suicide incidents, it is well-known that there is no significant difference from the self-report evaluation tools that solely evaluate the suicide incident. Moreover, the interview evaluation formula can eliminate the occurrence of errors due to misunderstanding of the evaluation contents, and the interviewer can evaluate the patient's suicide incidents with consideration of their education level and age group (57).

Fourth, in the case of the Korean version of the tools discussed above, many were developed or standardized only for specific groups. Even if the tool targets all adults, only college students, or just ordinary people, without validation across populations it seems unsuitable to use for the whole population. In order to be used nationwide, standardization based on a stratified sampling method that encompasses the clinical group, the general population, and various age groups is required. When using a nationally unified measurement tool, the indicators of suicidal ideation or behavior from different institutions, such as medical or educational organizations, can be matched and aligned to facilitate communication between such institutions, thereby allowing for the development of unified treatment and prevention guidelines across Korea.

Lastly, without reviewing whether the measurement tool reflects various social, psychological, and cultural factors within Korea, there is a problem of focusing only on the literal translation or the reverse translation and its validation while failing to take a holistic approach with considerations of contextual factors. Hence, in order to develop an appropriate tool for the Korean populations, future research should focus on creating new evidence-based measures using data that analyzed the warning signs and risk factors indicated by suicide deaths in Korea.

In conclusion, the main instruments that have been translated in Korean and have reported the appropriate psychometric properties are CCSRS, Beck Scale for Suicide Ideation, SRSSIO, Suicide Risk Scale for Medical Inpatients, and Suicide Risk Scale for Medical Inpatients. Although these tools are the most appropriate ones to use for screening, it is important to use them for further improvement along with additional information from the participants' families or acquaintance to supplement these assessments.

## Author Contributions

I-CB, SJ, and HJJ have contributed to the manuscript of the paper. I-CB reviewed all the measurement tools and drafted the paper. SJ and HJJ edited and made additional remarks to the manuscript. EK, GL, and DL helped to establish an assessment tool based on this review paper and reviewed the manuscript. All authors contributed to manuscript revision, read, and approved the submitted version.

## Conflict of Interest

The authors declare that the research was conducted in the absence of any commercial or financial relationships that could be construed as a potential conflict of interest.
